# Kimura's disease and ankylosing spondylitis: A case report

**DOI:** 10.1097/MD.0000000000021629

**Published:** 2020-08-21

**Authors:** Anping Chen, Beibei Cui, Yanhong Li, Qiuping Zhang, Mingqi Yuan, Yi Liu

**Affiliations:** a Department of Rheumatology and Immunology, West China Hospital, Sichuan University, Chengdu, Sichuan; b Key Laboratory of Hubei Provincial of Occurrence and Intervention of Rheumatic Diseases, Affiliated Minda Hospital, Hubei National University, Enshi, Hubei, China.

**Keywords:** ankylosing spondylitis, *ARPC1B* gene, cyclosporine, Etanercept, Kimura's disease

## Abstract

**Rationale:**

Ankylosing spondylitis (AS) and Kimura's disease (KD) which is quite rare are both chronic inflammatory diseases. Recently we encountered a patient who suffered from KD and AS, and some of his family members also suffer from AS. We, therefore, investigated this unique case and conducted the family-based whole exome sequencing to explore the possible genetic alterations.

**Patient concerns:**

Here, we reported a case of a 44-year-old Chinese man with multiple painless masses all over his body and a back pain for 32 years. His uncle and sister were diagnosed with AS.

**Diagnosis:**

The diagnosis of KD was based on the patient's clinical features and the biopsy of the neck masses. The diagnosis of AS was based on the patient's clinical features, HLA-B27(+) and the radiologic changes of sacroiliac joints. The genetic test showed that ARPC1B gene which was associated with recurrent infections, auto-inflammatory changes and elevated IgE levels was mutated in this patient.

**Interventions:**

Neck masses were removed by surgery. Systemic glucocorticoid, nonsteroidal anti-inflammatory agents, combined with cyclosporine were orally administered, and Etanercept was injected subcutaneously.

**Outcomes:**

The masses disappeared rapidly after surgery combined with systemic glucocorticoid, but relapsed shortly after the therapy was discontinued. Low dose glucocorticoid, cyclosporine and Etanercept could keep both KD and AS remained long-term remission.

**Lessons:**

Our experience suggests that low dose glucocorticoid, cyclosporine and Etanercept could be beneficial for the patient with KD and AS. The mutation of ARPC1B gene in this case, which is associated with immunologic disturbance, may increase the susceptibility of KD.

## 1 Introduction

Kimura's disease (KD), also known as eosinophilic lymphoid granuloma, is a rare chronic inflammatory disease.^[1]^ Most cases of KD are reported in middle-aged males in East and Southeast Asia, and the condition is characterized by painless subcutaneous masses predominantly in the head and neck region, and elevated peripheral eosinophil counts and serum IgE levels.^[2,3]^ The pathophysiology of KD remains unknown, but allergic reactions, trauma, and auto-inflammatory processes are all considered possible causes.^[4]^ To date no published studies have investigated the genetic background of the disease.

Many cases of autoimmune diseases complicated with KD have been reported, including systemic lupus erythematosus (SLE), Behcet's syndrome (BD), and IgG4-related diseases.^[4–6]^ To date, however, KD associated with ankylosing spondylitis (AS) has not been reported. Herein, we describe the case of a patient with familial AS who was diagnosed with KD. These cases suggest that KD may be more likely to occur in people with autoimmune or auto-inflammatory diseases. We surmise that in the current patient mutated genes may have contributed to the pathogenesis of KD by causing immune disorders.

## 2 Methods

### 2.1 Ethics statement and subjects

This study was approved by the West China Hospital Institutional Research Committee in accordance with the 1964 Helsinki declaration and its later amendments or comparable ethical standards. All subjects provided written consent to participate prior to the study. Written informed consent for publication was obtained from all participants.

A 44-year-old Chinese man presented with AS and KD, both of which had developed during childhood. His uncle had AS and had died many years ago. His father did not have AS and had died in an accident. His mother, his son, his sister, and himself all carried the HLA-B27 allele. His sister had been diagnosed with AS at 32 years old, but neither his mother nor his son had been diagnosed with the disease. The four family members were enrolled as subjects in the present investigation. The patient was admitted to the Department of Dermatology in West China Hospital of Sichuan University. Demographic and clinical data were collected from hospital records or questionnaires and reviewed by experienced physicians.

### 2.2 Genetic study methodology

EDTA anti-coagulated venous blood (5 mL) was collected from each of the four family members.

Genomic DNA was extracted using a DNA Extraction Kit (Tiangen Biotech, Beijing, China) in accordance with the manufacturer's protocol. Genomic DNA (3 μg) was fragmented via nebulization, and the fragmented DNA was end repaired and A-tailed using standard protocols. Illumina adapters were ligated to the A-tailed fragments, and the products were size-selected for a 350 to 400-base pair product. Whole exome enrichment was performed using the GenCap custom enrichment kit (MyGenostics Inc, Beijing, China) in accordance with the manufacturer's protocol. Biotinylated capture probes (80–120-mer) were designed to tile all of the exons with non-repeated regions and sequenced on an Illumina NextSeq 500 sequencer (Illumina, San Diego, CA) for paired-end reads of 150 bp.

Sequenced reads were aligned to the hg19 human reference genome sequence using BWA aln and BWA sample. Variations were called by GATK Haplotype Caller with default parameters, and after genotyping they were joined together by GATK Combine GVCFs/Genotype GVCFs. Variants were retained considering a read depth DP ≥ 8, MQ ≥ 20. Variants were annotated by ANNOVAR, filtered by position (non-synonymous or gain/loss of stops), VAF < 0.005 (1000 genome project [2012] and HAPMAP), and potential damaging effect (variants that were predicted to be damaging variants by at least two databases, including SIFT, PolyPhen2 HDIV, PolyPhen2 HVAR, LRT, Mutation Taste, Mutation Assessor, FATHMM, GERP++, PhyloP, and SiPhy).

## 3 Case report

### 3.1 Presenting patient

A 44-year-old Chinese man presented on September 11, 2018 with multiple painless masses all over his body for 33 years, and with back pain for 32 years. A small nodule had developed on his left post auricular region at the age of 21 years, followed by a similar nodule on his right post auricular region shortly thereafter. Masses appeared in the left inguinal lymph nodes, left epitrochlear lymph nodes, and neck during the last 10 years. At the age of 12 years he had begun to suffer from back pain that was more severe at night and could be relieved after activity, and he often took nonsteroidal anti-inflammatory agents spontaneously to relieve his pain. He denied to have fever, night sweats, edema, or weight loss. He had suffered from scleral ciliary body inflammation at the age of 31 years.

Physical examination revealed two soft to semi-firm infiltrated subcutaneous nodules in the regions of the right submandibular gland (3.5 cm in diameter) and left parotid gland (7.5 cm in diameter). Gaenslen's maneuver was positive, the Schober test result was 2 cm (<4 cm), and the cervical and lumbar range of motion was limited.

Laboratory investigations revealed consistently elevated serum IgE (320.3–936.02 IU/L, normal = 150 IU/L), eosinophilia (1.16–0.58 × 109/L, normal = 0.1–0.4 × 109/L), C-reactive protein (CRP; 20.5–4.1 mg/L, normal = 5 mg/L), and HLA-B27(+). Other parameters tested including hemoglobin, blood urea nitrogen, routine urinalysis, creatinine, urinary protein level, liver function, erythrocyte sedimentation rate, lactate dehydrogenase, auto-antibody spectrum, and rheumatoid factor were all within normal ranges.

**Figure 1:**
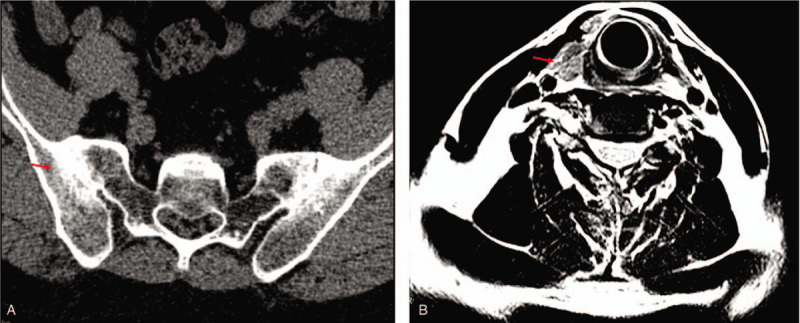
(A) Computed tomography scans of the sacroiliac joint depicted hyperosteogeny, bone destruction, joint space stenosis, and bony ankylosis. (B) Magnetic resonance imaging scans revealed subcutaneous masses on the left side of the neck.

**Figure 2:**
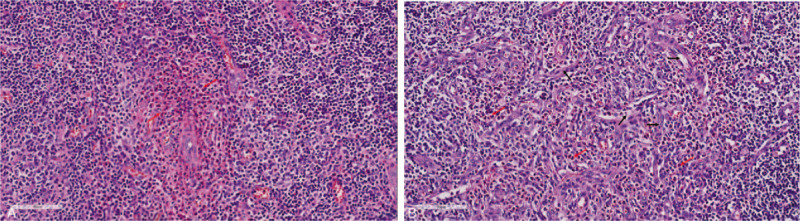
(A) Subcutaneous mass biopsy depicting eosinophilic infiltration (hematoxylin and eosin staining, ×100). (B) Subcutaneous mass biopsy also depicting extensive postcapillary venule proliferation and micro-abscesses (hematoxylin and eosin staining, ×100).

Computed tomography scans of the sacroiliac joint showed hyperosteogeny, bone destruction, joint space stenosis, and bony ankylosis (Fig. 1A). Magnetic resonance imaging depicted subcutaneous masses on the left side of the neck (Fig. 1B). An incisional biopsy of the neck mass revealed preserved lymph node architecture with follicular and focal paracortical hyperplasia. The interfollicular region contained scattered eosinophils and many endothelial venules. The perinodal fibroadipose tissue was extensively infiltrated by polymorphous inflammatory cells, including reactive lymphoid follicles, and numerous eosinophil that forming focal micro-abscesses (Fig. 2). Several treatments including systemic glucocorticoids, cyclosporin, and surgery had rapidly relief his symptoms, but he had relapsed shortly after therapy was discontinued. He underwent tumor removal at the ages of 15, 22, 34, and 36, respectively. Oral prednisone (0.5 mg/kg once a day) was given after each operation, and then gradually reduced. However, when the patients stopped hormone for half a year to one, the masses recurred. Oral prednisone (0.5 mg/kg once a day) and cyclosporine A (75 mg twice a day) was given at the age of 40, and then reduced to prednisone (5 mg once a day) and cyclosporine A (75 mg twice a day) for maintenance. The patient was followed up every 3 months. So far his masses have not relapsed. Anti-tumor necrosis factor inhibitors-Etanercept (25 mg once a week) was given at the age of 40. Under this treatment, his AS was relieved continuously.

### 3.2 Genetic analysis

We searched for genes that were mutated in the patient but not in his mother, sister or son, selected the genes with known disease associations, and screened for mutations in the normal population with a frequency >0.001 and a mutation rate <0.3. We surmised that the patient may have had more mutated genes associated with immune abnormality or autoimmune diseases.

**Figure 3:**
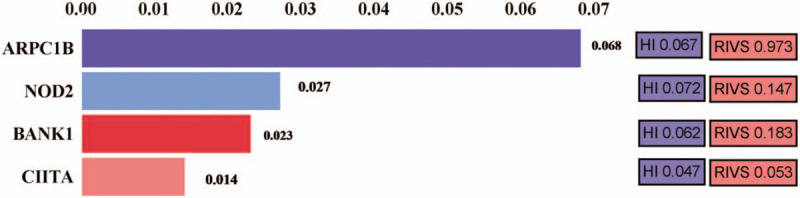
Gene prediction scores of the four genes and residual variation intolerance score of the genes.

The investigations revealed that ∼54 (Table 1) genes were mutated in patient but not in his mother, sister, or son. Of these, 25 were genes that may be associated with autoimmune or auto-inflammatory diseases or immune abnormality (Table 1). We used the OMIM database and Gene Cards database to further interpret these genes and found that *APC1B*, *NOD2*, *BANK1*, and *CIITA* genes which associated with immunologic derangement or autoimmune diseases may have some relationship to the predisposition of KD (Fig. 3). But none of them had been reported before. Phenolyzer were found that these four genes were in the same biosystem in the record of NCBI's Biosystem. According to the result of residual variation intolerance score (RVIS),^[7]^ ARPC1B had a RVIS score of-1.66 and a percentile of 2.72%, showing that it was amongst the 2.72% most intolerant of human gene (FDR = 8.11 × 10^−6^), the normalized RVIS of ARPC1B was 0.973 approximated to 1, indicating that this gene were considered as “intolerant” and had a HI score of 0.607, suggesting the haplo-insufficiency of it (Fig. 3).^[8]^

**Table 1:**
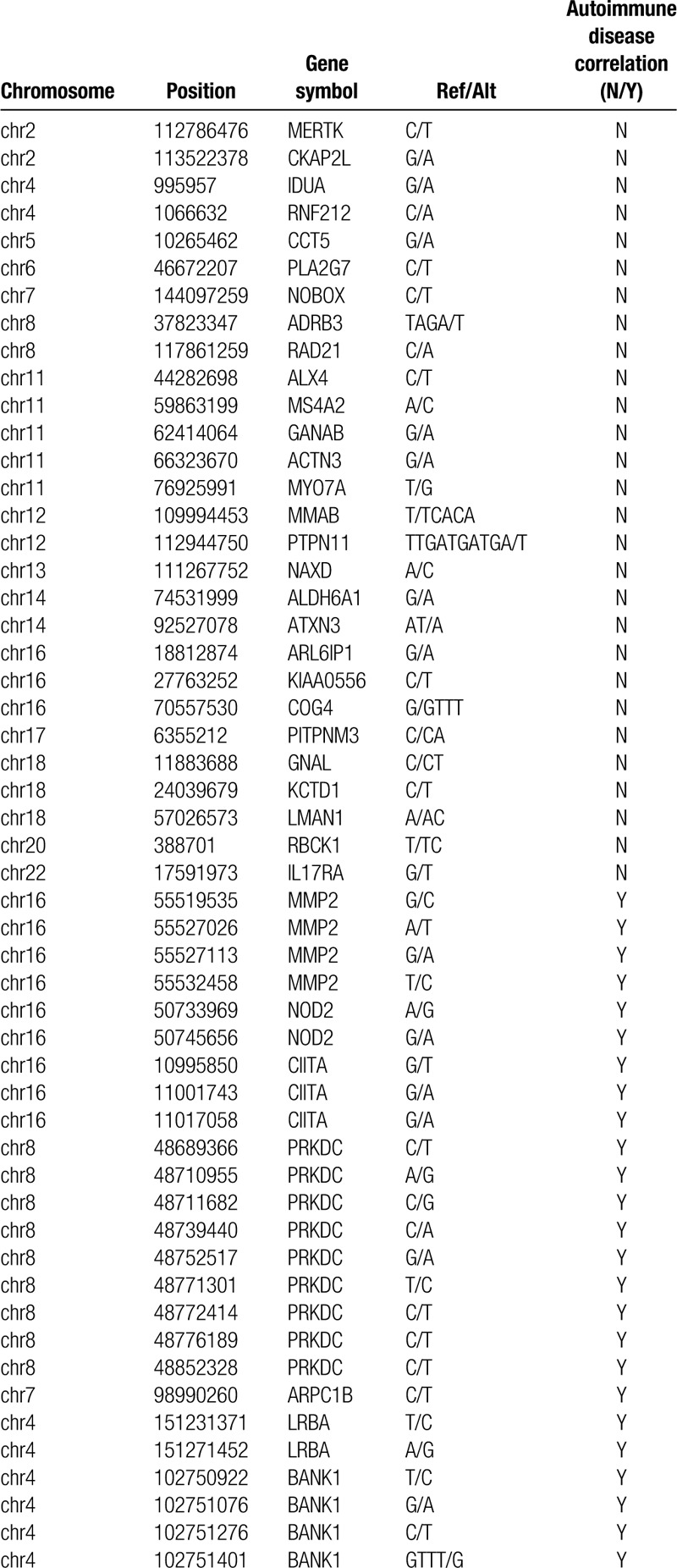
List of 54 genes with known diseases mutated in the patient by whole exome sequencing, and the 25 genes associated with autoimmune or autoinflammatory diseases or immune abnormality.

## 4 Discussion

This report describes a rare case of a patient who developed KD in combination with AS. Familial AS was also present in the current case. To date there have been no reports of the co-occurrence of KD and AS in one person and in the same family. All the family members discussed in the current report were HLA-B27 positive except the patient's father, and the patient and his sister had been diagnosed with AS but his mother, father, and son had not. Recently many studies have reported that genetic associations and immune abnormality were both involved in the pathogenesis of AS. These discoveries include genes encoding cytokine receptors, transcription factors, signaling molecules and transport proteins.^[9,10]^ They suggest that CD8^+^ T cells and IL-17 producing CD4^+^ T cells are inherently involved in the pathogenesis of AS, and that T cell-associated immune abnormality is an important component of AS.^[11]^

KD is a rare disease of unknown etiology, and allergic reaction, trauma, and autoimmunity have all been suggested as possible causes.^[12]^ It is currently believed that KD is also partly due to T cell-associated immune disorder.^[13]^ Some investigators have reported elevated CD4^+^ T cells and CD4^+^/CD8^+^ ratios in KD, and such phenomena have also be reported in patients with AS.^[14,15]^ Ohta et al^[16]^ reported that the Th1/Th2 ratio was reduced while Tc1/Tc2 was increased in KD patients, although there was no significant difference in the numbers of CD4^+^ and CD8^+^ cells between patients with KD and control patients. We speculate that in the present patient with AS abnormality of T cell subsets affected the Th1/Th2 ratio, and his unusual immunological background led to changes in serum IgE and increased susceptibility to KD. T cell function in patients with concurrent AS and KD has remained undefined to date, and should be further investigated.

Although the MHC class I allele HLA-B27 confers the greatest genetic risk of AS, genome-wide association studies have revealed more than 60 additional genetic risk factors for the disease, including many variants of multiple non-HLA genes.^[16]^ Notably however, genes associated with susceptibility to KD remain unclear. Recently the co-presence of an increasing number of autoimmune diseases with KD has been reported. Wang et al^[5]^ reported a case of KD concomitant with lupus nephritis in a 72-year-old male patient. Ben-Chetrit et al^[4]^ described a family in which three siblings suffered from Behcet's disease and an additional brother suffered from KD. Shun-Neng et al^[12]^ described a patient with KD associated with necrotizing eosinophilic vasculitis who presented with recurrent peripheral arterial occlusive disease. There is more than one previous report of the co-presence of KD and IgG4-related disease.^[6]^ These reports suggest that KD may be more likely to occur in patients with autoimmune or auto-inflammatory diseases. We surmise that mutated genes leading to immune disorders in these patients may contribute to the pathogenesis of KD.

The involvement of genetic associations and immune pathways in the pathogenesis of AS has been widely confirmed.^[11]^ Whereas, to date, no studies have reported that genetic background is implicated in the pathogenesis of KD.^[17]^ Given the rarity of KD, its occurrence in the present young male with AS and the reported patients with other autoimmune diseases led us make a hypothesis that there may harbor some underlying genetic alterations associated with immune abnormality that made the susceptibility of KD. We compared the gene mutations between the patient and his family members who shared the HLA-B27 haplotype via whole exome sequencing, and found that he harbored more mutated genes that are associated with immune abnormality. According to the OMIM database and GeneCards database, the four genes *ARPC1B*, *NOD2*, *BANK1*, and *CIITA* are shown to be related to the predisposition of KD. The normalized RVIS of *ARPC1B* was approximated to 1, indicating that this gene was considered as “intolerant” genetic factor. Meanwhile, *APRC1B* had a HI score of 0.607,^[8]^ suggesting haplo-insufficiency of it. While *NOD2*, *BANK1*, and *CIITA* with positive scores had more common functional variation.

ARPC1B is a key factor in the assembly and maintenance of the ARP2/3 complex, which is involved in actin branching from an existing filament.^[18]^ Stefano et al^[19]^ analyzed 14 patients with biallelic ARPC1B mutations, and reported combined immunodeficiency, allergy, and auto-inflammation caused by the mutations in these patients. Clinical symptoms in these patients involved platelet abnormalities, eosinophilia, immune-mediated inflammatory diseases, and combined immunodeficiency. Abnormal immunoglobulin levels were observed in vivo, with markedly increased IgA and IgE in almost all cases. KD is also characterized by elevated peripheral eosinophils and serum IgE, and it is considered an inflammatory disease leading to type I hypersensitivity reaction or auto- immune disorder.^[20]^ Brigida et al ^[15]^ demonstrated that *ARPC1B* deficiency may also lead to cytoskeleton and functional defects in T cells, but notably the patient in the present case was not biallelic for *ARPC1B* mutations, thus, the *ARPC1B* mutation in this patient may have been partially involved in the pathogenesis of KD.

In conclusion, this is the first report of the co-presence of KD and AS in the same patient, and in a patient with a genetic familial history of AS. Family-based whole exome sequencing determined that 54 genes were mutated in the patient, and 25 genes of them may be associated with autoimmune or auto-inflammatory diseases or immune abnormality. Among these 25 genes, *ARPC1B* which are reportedly associated with immunologic derangement or autoimmune disease may also be associated with a predisposition to KD. But further large-scale studies are required to investigate the functional role of these genes in KD, especially in KD complicated with AS.

## Acknowledgments

We thank Dr Owen Proudfoot from Liwen Bianji, Edanz Editing China (www.liwenbianji.cn/ac) for editing the English text of a draft of this manuscript.

## Author contributions

YL contributed to the conception of this case. AC and BC contributed significantly to analysis and wrote the manuscript. YL and MY contributed to the collection of clinical dates. QZ contributed to the genetic analysis.

**Conceptualization:** Anping Chen.

**Data curation:** Anping Chen, Beibei Cui.

**Formal analysis:** Anping Chen, Beibei Cui.

**Funding acquisition:** Yi Liu.

**Methodology:** Qiuping Zhang.

**Project administration:** Yi Liu.

**Resources:** Mingqi Yuan.

**Writing – review & editing:** Yanhong Li.
